# Are visual functions diagnostic signs of the minimally conscious state? an integrative review

**DOI:** 10.1007/s00415-018-8788-9

**Published:** 2018-02-28

**Authors:** Berno U. H. Overbeek, Henk J. Eilander, Jan C. M. Lavrijsen, Raymond T. C. M. Koopmans

**Affiliations:** 1 Department of Primary and Community Care, Centre of Family Medicine, Geriatric Care and Public Health, Radboud University Medical Centre, Nijmegen, PO Box 9101, 6500 HB Nijmegen, The Netherlands; 2 Azora, PO Box 30, 7060 AA Terborg, The Netherlands; 3 Kalorama, PO Box 85, 6573 ZH Beek, The Netherlands

**Keywords:** Disorders of consciousness, Minimally conscious state, Visual pursuit, Visual fixation

## Abstract

Visual pursuit (VP) and visual fixation (VF) have been recognized as the first signs of emerging consciousness and, therefore, are considered indicative of the minimally conscious state (MCS). However, debate exists about their status as they are considered either conscious reactions or reflexes. The aim of this study is to review the evidence of the definition, operationalization, and assessment of VP and VF in unconscious patients. PubMed and EMBASE were searched for relevant papers between May 26, 1994 and October 1, 2016. In addition, an internet search was done to identify other relevant papers, reports and manuals of assessment methods. Papers were included if the definition, operationalization, or assessment method of VP and VF was discussed in patients with disorders of consciousness. We identified 2364 articles, of which 38 were included. No uniform definitions of VP and VF were found. VP and VF were operationalized differently, depending on which scale was used. The Coma Recovery Scale-revised and the Sensory Tool to Assess Responsiveness were the only diagnostic scales found; the other scales were developed to monitor DOC patients. The use of a mirror was the most sensitive method for detecting VP and VF. The literature about the importance VP and VF in relation with consciousness is controversial. This integrative review shows a lack of consensus regarding the definition, operationalization, and assessment of VP and VF. International consensus development about the definition, operationalization, and assessment of VP and VF is recommended.

**Electronic supplementary material:**

The online version of this article (10.1007/s00415-018-8788-9) contains supplementary material, which is available to authorized users.

## Introduction

The unresponsive wakefulness syndrome, previously named vegetative state (UWS/VS) [1], and the minimally conscious state (MCS) are one of the worst possible outcomes of acquired brain injury. Patients in UWS/VS show no signs of consciousness [2], whereas MCS patients demonstrate minimal signs of consciousness such as following simple commands, gestural and/or verbal yes/no responses, intelligible verbalization, and purposeful behavior [3]. Complexity of behavior varies between MCS patients; therefore, subcategorization into MCS − (minus) and MCS+ (plus) was proposed. Patients in MCS − only demonstrate non-reflex behavior, whereas MCS+ patients demonstrate command following [4]. Differentiating between UWS/VS and MCS is difficult, as demonstrated by misdiagnosis rates of around 40% [5–8]. A correct diagnosis of MCS is important for several reasons. First, prognosis is more favorable compared to UWS/VS. A follow-up study showed that improvement beyond a year was absent in UWS/VS patients, whereas 1/3 of MCS patients emerged to consciousness beyond a year [9]. Second, MCS patients might have pain perception capacity, which has consequences for pain management [10]. Third, MCS patients have better outcomes from early intensive neurorehabilitation [11–13], recently confirmed in a long-term follow-up study [14]. Fourth, MCS patients may benefit from promising treatment options such as deep brain stimulation [15, 16] and pharmacologic therapies [17–19]. Compared to UWS/VS, other ethical dilemmas may arise in MCS patients, e.g., regarding suffering or withdrawing or withholding medical treatment [20].

Currently, an accurate diagnosis of MCS is based on behavioral assessment. Techniques, like neuroimaging, have not been implemented in clinical practice yet. Visual pursuit (VP), which has also been described as visual tracking,^1^ and visual fixation (VF) are considered the first signs of emergence of consciousness [21, 22]. According to the Coma Recovery Scale-Revised (CRS-R), which is the most used assessment scale, VP and VF are clinical signs denoting MCS [23]. According to the CRS-R, VP is present when a moving mirror is followed for 45° without loss of fixation in two of four directions, whereas VF is present when the eyes move from the initial fixation point and re-fixate more than 2 s in two of four trials [24].

Previously, it was demonstrated that failure to detect VP and VF caused misdiagnosis of MCS [7]. This was confirmed in a Dutch prevalence study about UWS/VS [8]: 39% of the reported UWS/VS were misdiagnosed and were at least in MCS. In the 15 MCS patients, VP was seen in 8 of them and VF in one. It remains subject of debate whether or not VP and VF are clearly discernible signs of consciousness. In 1994, the Multi-society Task Force on Persistent Vegetative State (MSTF) reported that VP and VF could be either considered as signs of consciousness or as brief visual orienting reflexes. The MSTF advised to be cautious in diagnosing UWS/VS if VP and/or VF are observed [2]. In 1996, an International Working Party doubted the relation of VP with the conscious state, considering the sole presence of VP not as a reliable sign of consciousness [25, 26]. In 2002, the definition and diagnostic criteria for MCS were published [3]. These criteria were consensus based due to the lack of scientific evidence about diagnosis and prognosis of MCS. VP was incorporated into the criteria as it was considered an example of purposeful behavior based on the following data: VP was associated with late improvement [27], more prevalent in MCS patients [21], and preceded interactive and social behavior later in the recovery course [28]. Regarding the incorporation of VF into the criteria of MCS, no supporting data were reported. Currently, the question whether VP and VF are signs of consciousness still remains debatable. However, in daily practice and in the most recommended assessment scale [23, 24], VP and VF are considered important signs of MCS.

To determine if VP and VF are indicative of consciousness, data about their diagnostic validity are necessary. In 2014, a review about eye movement measurement in the diagnostic assessment in disorders of consciousness (DOC) [29] focused on quantitative techniques to measure eye movements rather than on behavioral assessment. However, this review did not address the question whether VP and/or VF are diagnostic signs of consciousness.

The aim of this study is to review the evidence about definition, operationalization and assessment of VP and VF in relation with the state of consciousness.

## Methods

We performed an integrative review, which provides a comprehensive understanding of a particular phenomenon or healthcare problem. This method has the possibility to include a variety of data [30, 31].

### Search strategy

The databases of PubMed and EMBASE were searched from May 26, 1994 until October 1, 2016. The publication of the position paper of the Multi-society Task Force on the Persistent Vegetative State was chosen as start date, because they discussed the significance of VP and VF for both UWS/VS and higher levels of consciousness [2]. We searched on the internet for guidelines, reports and for manuals of assessment scales and searched the websites of international taskforces on DOC for relevant papers. The bibliographies of the selected articles were searched for additional relevant papers. Searches were limited to the English, German, French, and Dutch languages.

Two search strategies were used: a broad, general search regarding diagnosis and prognosis in DOC patients and a more specific search related to the use of VP and VF in the diagnosis of DOC.

For the broad general search, we combined patient-related terms like ‘persistent vegetative state’ and ‘minimally conscious state’ with a diagnostic filter and the terms ‘misdiagnosis’, ‘assessment’, and ‘prognosis’. For the specific search, we combined the previously mentioned patient-related terms with terms like ‘visual pursuit’, ‘visual tracking’, ‘visual fixation’, ‘visual perception’, and ‘vision disorders’. Finally, we combined the results of the broad and the specific searches (Supplement 1).

### Study selection

Papers were selected if they met one or more of the following inclusion criteria: (1) VP and VF were discussed, either described as DOC in general or described as UWS/VS and/or MCS; (2) the etiology of UWS/VS and MCS was brain injury caused by an acute incident; (3) discussion of the operational definition of VP and/or VF; (4) discussion of different assessment methods; (5) use of an assessment scale testing VP and/or VF; (6) discussion of assessment items of either VP and/or VF; and (7) discussion of influencing factors on visual responses in the assessment of DOC. Papers were excluded if DOC was caused by neurodegenerative diseases and if VP and VF were discussed in patients without DOC.

### Data extraction and validation

The first author (BO) reviewed the papers. In case of doubt, a second reviewer (HE) was consulted. After discussion, a decision about inclusion was reached by consensus.

Before reviewing all citations, agreement about the inclusion and exclusion criteria was investigated. Two researchers (BO, HE) independently screened a sample 200 titles and abstracts. After extracting 2 duplicates, 198 papers were checked. Agreement about direct inclusion or papers eligible for further analysis of full text was reached in 168 (85%) of the papers.

Since disagreement existed about a considerable number of papers (n = 30, 15%), we added another search strategy. If based on title and abstract no decision could be made, the full text was electronically screened with the term ‘visual’ to find the terms ‘visual pursuit’, ‘visual fixation’, and ‘visual tracking’. If one of these items was discussed in patients with DOC, the article was eligible for screening of the full text. If not, the paper was excluded. Reanalysis of the 30 papers resulted in disagreement in 2 papers. Thus, adding this method to the search strategy decreased disagreement from 15 to 1%. Disagreement about inclusion was resolved through discussion between both reviewers, which led to consensus.

The selected papers were analyzed by the first author with a data extraction form. This form contained information about: type of article, aim, study subjects, outcome measures, main results, and conclusions.

## Results

### Included studies

**Fig. 1:**
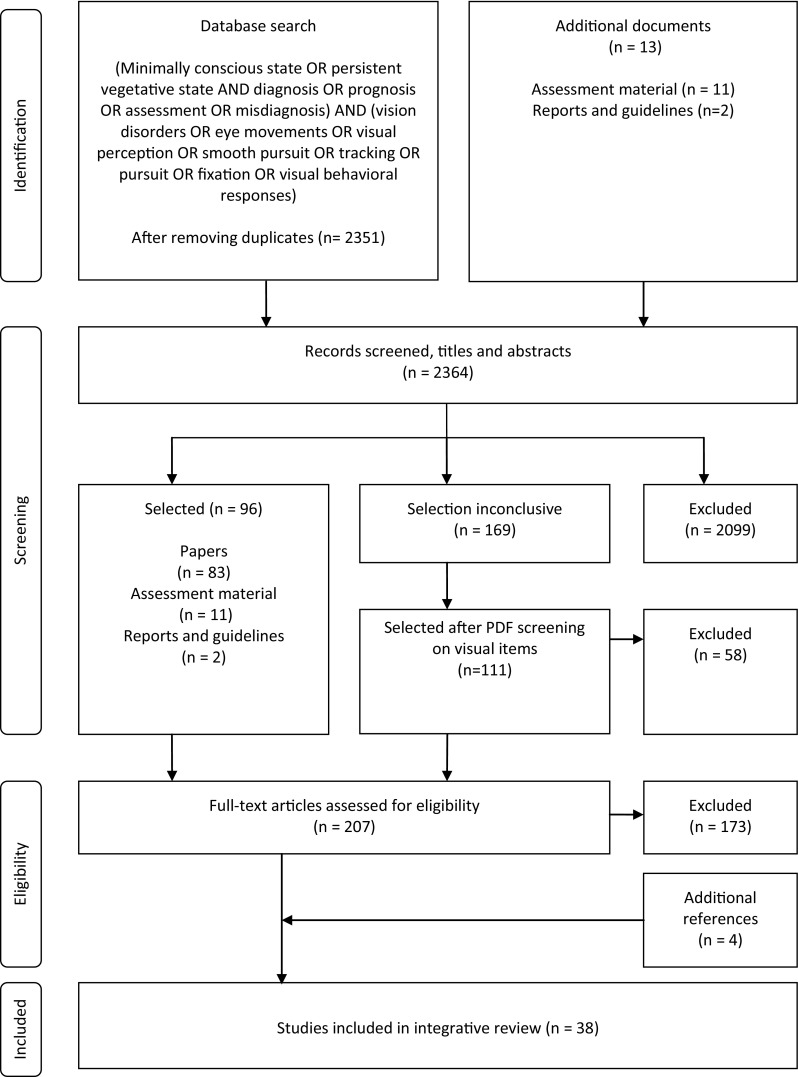
Flow diagram

Through the database search, 2351 papers and 13 additional documents were found (Fig. 1). After screening all titles and abstracts, 96 papers and documents were selected for full text analysis. No decision based on title and abstract could be made for 169 papers. Electronic full text screening of these papers yielded 111 eligible for further analysis. In total, full text of 207 papers was analyzed. Eventually, 34 papers could be included. After manual searching the bibliographies of the selected papers, four additional papers were included. The final sample consisted of 38 papers.

### Definition

Descriptions of VP and VF were found in six papers; however, no uniform definitions of VP and VF were found. The papers provided eight descriptions of VP and 3 of VF [3, 25, 26, 32–34] (Table 1). VP was denoted by the terms eye tracking, tracking eye movements, horizontal and vertical tracking and pursuit eye movements [3, 25, 26, 32, 34]. VP was described as following objects or people [25, 26], as localizing to a visual stimulus [32], as the ability to follow in the horizontal and visual fields [32], and as a reaction to a moving stimulus [3]. VF was denoted by eye contact which was further explained as the patient’s gaze during the majority of the assessment session [32], as sustained fixation in response to a salient stimulus [3], and as active looking at or for objects [33].

**Table 1:** Descriptions of visual pursuit/visual tracking and visual fixation

Author and year [references]	Visual response^a^	Descriptions (quotes from original text)
Andrews 1996, report of International Working Party on the Management on PVS [26]	Visual tracking	“Eye tracking is when a patient follows a moving object by moving the eyes”
Andrews 1996, summary of report International working Party Management on PVS [25]	Visual tracking	“Tracking eye movements following objects or people”
Ansell et al. 1989 [32]	Eye tracking	“Eye tracking: localizing to a visual stimulus”
	Horizontal tracking	“Horizontal tracking: ability to follow visually through left and right visual fields”
	Vertical tracking	“Vertical tracking: ability to follow visually through upper and lower visual fields”
	Eye contact	“Eye contact: patient’s gaze during the majority (50%) of the session”
		“Eyes focussed on the examiner (50% or more)”
Giacino et al. 2002 [3]	Pursuit eye movements	“Pursuit eye movements or sustained fixation that occur in direct response to moving or salient stimuli.”
	Sustained fixation	
Rader and Ellis 1994 [34]	Visual tracking < 3 s	“Eye movements toward stimulus (patient appears to be “looking at” stimulus and/or stimulator) for less than 3 s.”
	Visual tracking > 3 s	“Eye movements toward stimulus (patient appears to be “looking at” stimulus and/or stimulator) for more than 3 s.”
Wade and Johnston 1999 [33]	Visual fixation	“···visual fixation active looking at or for objects”

*PVS* persistent vegetative state

^a^Terminology used by the authors

### Assessment and operationalization of VP and VF

Assessment and operationalization of VP were found in 14 papers in which 9 assessment scales were discussed [23, 24, 28, 32, 34–43] (Table 2). Another scale, the Sensory Modality Assessment Rehabilitation Technique (SMART) was identified [44], but could not be included, since this scale was not available for evaluation. The assessment scales were developed with different purposes and have different testing procedures and variable operational criteria. Scales with a diagnostic purpose are the CRS-R and the Sensory Tool to Assess Responsiveness (STAR) [23, 24, 43]. In these scales, VP indicates MCS. Scales with purposes of detecting and monitoring signs of consciousness are the Western Neuro Sensory Stimulation Profile (WNSSP) [32], Disorders Of Consciousness Scale (DOCS) [35], Loewenstein Communication Scale (LCS) [36], Comprehensive Assessment Measure for Minimally Responsive Individuals (CAMMRI) [37, 38], Sensory Stimulation Assessment Measure (SSAM) [34], Coma Near Coma Scale (CNC) [39, 40] and the Wessex Head Injury Matrix (WHIM) [28, 41, 42]. VP was tested with different stimuli: objects [28, 32, 34–38, 41, 42], pictures and/or photographs [32, 35, 37, 38], mirror [23, 24, 32, 35, 37, 38, 43], and an individual [28, 32, 34, 36, 39–42]. In the CRS-R [23, 24], VP was operationalized as following a mirror without loss of fixation in 2/4 trials. In the STAR [43], VP is operationalized slightly different from the CRS-R, the number of trials which is 4 compared to 2 in the CRS-R and the duration of fixation on the mirror is set on 2 s or longer. In the Wessex Head Injury Matrix (WHIM) [28, 41, 42], VP is tested in four reactions, which each have a separate operational definition. A reaction is present if the observed reaction is in accordance with the operational definition of the reaction. The other scales score VP by rating the observed reactions with points [32, 34–40].

**Table 2:** Assessment and operationalization of visual pursuit/visual tracking

Author and year [references]	Assessment method	Purpose of method	Method of testing	Operationalization
Ansell et al. 1989 [32]	WNSSP	Measuring cognitive and communicative function in severely head injured patients	Present mirror, picture, object in midline position Slowly move item from left to right across midline. Repeat several times, if necessary Use colored pictures, preferentially family pictures. Use bright objects or objects with moving parts Tracking of individual: walk to the opposite side of the patient’s bed Test tracking of all stimuli in horizontal and vertical planes	*Scoring system* (*points*) Horizontal tracking: 0: no response 1: following from midline to 1 side 2: following from midline to 2 sides 3: following across midline Vertical tracking: 0: no response 1: following in 1 direction 2 following in 2 directions
Bender Pape 2011 [35]	DOCS	Measuring neurobehavioral functioning during coma recovery	Present 3D objects, familiar faces picture and mirror Present in left visual field and slowly move across midline to right visual field, and vice versa Present 3D object/familiar face in middle visual field and slowly move upward and downward Present each item as many times as necessary to determine best response Test in horizontal and vertical planes	*Scoring system* (*points*) 2: localized response. Visual orientation toward object Separate scores for each visual field. Score 2 if tracking in at least one visual field. If 2 is not scored, have subject track themselves with a mirror Separate scores for each visual field
Borer et al. 2002 [36]	LCS	Provide information about communicative abilities in minimally responsive patients and indicator of rehabilitation potential	Object, people^a^ Vertical and horizontal planes	*Scoring system* (*points*): 0: no tracking 1: inconsistent selective tracking 2: consistent selective tracking 3: diminishing tracking 4: constant qualitative tracking
Giacino et al. 2004 [23], Giacino and Kalmar 2004 [24]	CRS-R	Diagnostic distinction between UWS/VS and MCS	Mirror, 4-6 inches in front of face, verbally encourage patient to fixate on mirror, move 45° to left, right, up, down Repeat procedure, total of 2 trials in each direction administered	*Response scored if:* Eyes must follow mirror for 45° without loss of fixation on 2 occasions in any direction.
Gollega et al. 2014, 2015 [37, 38]	CAMMRI	Detection of subtle signs of consciousness	Pictures/photographs, mirror, target stick (circle mounted on a stick). Up to three stimuli can be presented Instruct client to look at stimulus, place 18 inches away from eye, tell client to keep looking at moving target. If no tracking observed with one stimulus, try another stimulus. Horizontal and vertical: slight arc of 45° from midline to left/right and up/down, respectively Diagonal tracking: test only if at least partial horizontal and vertical tracking are observed, move stimulus slowly diagonally. Start in visual quadrant that showed the best tracking Do 3 tests for each plane	*Scoring system* (*points*): Fixes gaze: 0: no fixation, 1: fixation Horizontal and Vertical Tracking 0: no tracking, 1: partial tracking in left, right, upper or lower field (1 point for each field)^b^ 2: full tracking in left, right, upper or lower field (2 points for each field)^c^ Diagonal tracking 0: no tracking 1: tracking
Rader and Ellis 1994 [34]	SSAM	Measuring the unconscious patient for a long period over time	Separate assessor and rater looking at stimulus or stimulator	*Best response to stimulation recorded, points scored* 1: eye movement not different from baseline 2: eye opening in response to stimulus 3: visual tracking < 3 s: Eye movement toward stimulus, patient appears to be “looking at” stimulus or stimulator less than 3 s 4: visual tracking > 3 s: Eye movement toward stimulus, patient appears to be “looking at” stimulus or stimulator more than 3 s 5: blinks, opens, or closes eyes in response to command 6: answer to simple questions by eye movements
Rappaport 1990 [39], Rappaport et al. 1992 [40]	CNC	Detection of small changes in neurobehavioral status in patients in UWS/VS or near-vegetative states	Tell patient “look at me” move your face 20 inches away from side to side Horizontal plane 5 trials	*Scoring system (points)* 0: sustained tracking (at least 3x) 2: partial tracking (1–2×) 4: no tracking
Stokes et al. 2016 [43]	STAR	Graded assessment of motor, sensory and communicative responses to sensory programme	Mirror Hold a mirror in front of the patient, move to all four quadrants of visual field. Horizontal, vertical Repeat 3 times (4 trials in total), with a ten second delay after each trial.	Sustained visual pursuit showing localization response: Fixates on mirror for at least two seconds, at least twice, during the four trials
Shiel et al. 2000 [28, 41, 42]	WHIM	Monitoring changes from coma to consciousness	*Behavior observed* Item 12: eyes follow person moving in line of vision	*Operational definition* Eyes move in direction of person, from midline to either left or right
			*Behavior observed* Item 16: turns head to person who is talking	*Operational definition* Moves eyes or turns head at person
			*Behavior observed* Item 17: watches person moving in line of vision. Person moves from one side to other side of the bed	*Operational definition:* Eyes follow from end of bed to left or right or both.
			*Behavior observed* Item 18: tracks for 3–5 s. Attract patient’s attention with a brightly colored object, move through the visual field	*Operational definition* If patient tracks through at least 90 degrees

^a^No assessment instruction found

^b^Tracking of part of visual field

^c^Tracking of entire visual field

VF was assessed and operationalized in 12 papers, which discussed seven assessment scales [23, 24, 28, 32, 35–42] (Table 3). Testing procedures and operationalization varied between the scales. The only scale with a diagnostic purpose is the CRS-R [23, 24]. In this scale, VF indicates MCS. Scales with purposes of detecting and monitoring signs of consciousness are the WNSSP [32], DOCS [35], LCS [36], CAMMRI [37, 38], CNC [39, 40] and the WHIM [28, 41, 42]. VF was tested with different stimuli: an individual [28, 32, 37, 38, 41, 42], pictures of familiar faces [28, 35, 37, 38, 41, 42], brightly colored or illuminated objects [23, 24, 37, 38], a mirror [37, 38], objects [28, 37, 38, 41, 42] and light flashes [39, 40]. The CRS-R operationalizes VF as re-fixation on an object 2 s or longer and indicates MCS [23, 24]. In the WHIM, 8 reactions test VF, each reaction has its own operational definition and VF is considered present if the operational definition is met [28, 41, 42]. The other scales score VF by rating to different observed reactions with points [32, 35–40].

**Table 3:** Assessment and operationalization of visual fixation

Author and year [references]	Assessment method	Purpose of method	Method of testing	Operationalization
Ansell et al. 1989 [32]	WNNSP	Measuring cognitive and communicative function in severely head injured patients	Observation of focusing of patient on the examiner	*Scoring system* (*points*) 0: eyes closed, 1: eyes open, not focused on the examiner, 2: eyes focused on examiner (50% or more), 9: unable to open the eyes (CN III paralysis)
Bender Pape 2011 [35]	DOCS	Measuring neurobehavioral functioning during coma recovery	Focus on familiar face. Hold picture of familiar person to patient for at least 1 year prior to injury approximately 18 inches to face for 5–10 s. Test in upper, lower, middle, left and right visual field	*Scoring system* (*points*) 2: localized response. Visual orientation toward object. Separate scores for each visual field Score 2 if focusing on familiar face in at least one visual field
Borer-Alafi et al. 2002 [36]	LCS	Provide information about communicative abilities in minimally responsive patients and indicator of rehabilitation potential	Gaze is observed^a^	*Scoring system* (*points*): 0: no use of the visual channel: closed eyes, no eye movements, no papillary response to stimuli, et cetera 1: congealed, freezed gaze 2: aroused look, eye movements apparently directed to the environment 3: inconsistency in focusing on stimuli 4: aroused look with consistent focus on stimuli
Giacino et al. 2004 [23], Giacino and Kalmar 2004 [24]	CRS-R	Diagnostic distinction between UWS/VS and MCS	Brightly colored or illuminated object presented in front of patient’s face, readily move to upper, lower, right, left visual fields Horizontal and vertical plane 2 trials in each plane	*Scoring criteria* Eyes move from initial fixation point and re-fixate at least 2 s on new target. At least 2 episodes of fixation are required
Gollega et al. 2014,2015 [37, 38]	CAMMRI	Detection of subtle signs of consciousness	*Various stimuli:* pictures/photographs, mirror, target stick (circle mounted on a stick) Position yourself so that you can present the stimulus in the client’s visual midline Tell client you have something for him to see. Ask him to try and do his best Place target where eye appears to look and 18” away from eye Move target away 6” or more where target was. Observe if the eye(s) look at the target and note for how long (less than 2 s or at least 2 s) Try to use visual targets of interest. Up to three stimuli can be presented. Do 4 tests: up/downward, left/right	*Scoring system* (*points*) Score 0: no fixation in any of the 4 trials, discontinue visual response testing (do not test visual tracking) Score 1: If client fixates gaze, score 1 and proceed with visual tracking test
Rappaport et al. 1992 [39, 40]	CNC	Detection of small changes in neurobehavioral status in patients in UWS/VS or near-vegetative states	Light flashes 1 per second, in front, slightly left, right up and down, each trial 5 trials	*Scoring system (points)* 0: sustained fixation or avoidance (at least 3×) 2: partial fixation (1–2×) 4: none
Shiel et al. 2000 [28, 41, 42]	WHIM	Monitoring changes from coma to consciousness	*Behavior observed* Item 5: looks at person briefly	*Operational definition:* Eyes move aimlessly but remain on object/person when noticed. Briefly: impression of looking at
			*Behavior observed* Item 8: makes eye contact. Stand where patient is not directly seeing you, call patient’s name	*Operational definition:* Patient switches gaze and maintains eye contact for at least 3 s
			*Behavior observed* Item 9: patient is looking to person who is talking to them	*Operational definition* Gaze switch to person who is talking, at least for 3 s
			*Behavior observed* Item 13: looks at person giving attention	*Operational definition* Eyes rest at least 3 s on person giving attention
			*Behavior observed* Item 24: maintains eye contact for ≥ 5 s	*Operational definition* Looks at person for 5 s or longer
			*Behavior observed* tem 28: looks at object when requested, hold brightly colored object out of view, ask patient to look at object	*Operational definition* Holds up brightly colored object out of patients immediate view and ask patient to look at it
			*Behavior observed* Item 33: seeks eye contact	*Operational definition* Moves head or eyes to make eye contact and maintains this ≥ 3 s
			*Behavior observed* Item 35: looks at/explores pictures etc.	*Operational definition* Pictures, photographs etc.: looks to, puts down, looks at another picture, etc.
			*Behavior observed* Item 36: switches gaze from one person to another	*Operational definition* Two people present in room positioned so that patient’s eyes must move or head must be turned to switch gaze from one person to the other. Spontaneously looks from one person to another

^a^No information given about testing procedure

### Assessment of visual pursuit

Assessment of VP was discussed in seven papers [45–51] (Table 4). Results were found about the direction of tracking [45, 47], time of assessment [46], different stimuli [47, 50, 51], quantitative assessment with an eye tracker device [48], and the use of personalized stimuli [49].

**Table 4:** Assessment of visual tracking/visual pursuit

Author, year [references]	Assessment item	Population	Assessment method	Results of study	Conclusion of authors
Ansell 1995 [45]	Direction of tracking	Closed head injury (*n* = 76)	WNSSP	48% showed preference: 28% preference of tracking in horizontal plane, 20% in vertical plane No significant differences in tracking preference between patients who recovered and those who did not	Individual preferences for plane of tracking, no group effects
	Type of stimulus		WNSSP 4 stimuli Mirror Individual Meaningful picture Object (snow globe)	Mean visual scores (points) Mirror 36.4 Individual 17.0 Picture 21.3 Object 18.7	Patients who recovered to consciousness had higher visual tracking scores on the WNSSP when a mirror was used
Candelieri et al. 2011 [46]	Time of assessment	UWS/VS (*n* = 9), MCS (*n* = 13)	CRS-R	Highest probability of observing visual tracking: 10.30 AM and 3.00 PM, lowest probability of detecting visual tracking: 2.00 PM	Time of assessment influences probability of detecting visual tracking
Thonnard et al. 2014 [47]	Type of stimulus	MCS (*n* = 88)	CRS-R	Patients tracked mirror (97%) over person (69%) and object (57%)	Majority of patients showed visual tracking when mirror was used
	Direction of VP	MCS- (*n* = 47), MCS + (*n* = 47)	CRS-R	Entire group: significantly more horizontal (*n* = 80) than vertical tracking (*n* = 61) MCS-: significantly more horizontal (*n* = 41) than vertical tracking (*n* = 35) MCS + : no significant difference between horizontal and vertical tracking. Chronic patients: significantly more horizontal (*n* = 61) than vertical tracking (*n* = 47)	Patients in MCS showed preferential horizontal visual pursuit compared to vertical visual pursuit
Trojano et al. 2012 [48]	Quantitative assessment	UWS/VS (*n* = 9), MCS (*n* = 9)	Infrared eye tracker: Visual pursuit defined as series (bouts) of fixations Images of parrot or circle	On-target fixations: UWS/VS 4.9% (below chance level of 10%) MCS 32.9%	Proportion of on-target fixations significantly differentiated MCS from UWS/VS, whereas mean duration of fixation bouts did not
Trojano et al. 2013 [49]	Personal relevant stimulus	UWS/VS (*n* = 13), MCS (*n* = 13)	Infrared eye tracker Images of parrot or circle or face of relative	MCS: significant higher % of on-target fixations (37.3%) when looking at relative’s face compared to circle (29.9%) and parrot (30.6%)	Higher percentage of tracking to a personal relevant stimulus
Turner-Stokes et al. 2015 [50]	Person/object	UWS/VS (*n* = 12) MCS- (*n* = 12) MCS + (*n* = 15) Emerged (*n* = 26)	WHIM	*Observed reaction* *state of consciousness* % Eyes follow person moving in line of vision MCS minus VS 1%; MCS- 38%; MCS + : 73% Tracks brightly colored object for 3-5 s: MCS minus VS 1%; MCS- 24%; MCS + 59% Watches person moving in line of vision MCS minus VS 0%; MCS- 27%; MCS + 63%	No conclusion^a^
Vanhaudenhuyse et al. 2008 [51]	Type of stimulus	MCS (*n* = 51)	CRS-R	Detection of visual tracking in individuals who showed visual tracking: mirror 95%, person 66% and object 55%, only tracking mirror 29%	More than a fifth of the patients only tracked a mirror (and not a moving person or object)

^a^No conclusion drawn about visual pursuit/visual tracking, data derived from Table 2

Regarding direction of tracking, 48% of 76 head injured adults showed a tracking preference: 28% in the horizontal fields and 20% in the vertical fields [45]. Another study investigated the tracking preference in MCS patients and showed that the MCS- group had a preference of tracking in the horizontal field whereas in MCS + no tracking preference was found [47].

Individual variability of VP within the day was investigated and the highest probabilities for detecting VP were seen at 10.30 AM and at 3.00 PM. The lowest probability for detecting VP was at 2.00 PM, being a post-prandial time point [46].

The use of a mirror was the stimulus with the highest scores in DOC patients. In 1995, it was demonstrated that patients following a mirror had significantly higher mean scores on the visual tracking scale of the WNSSP compared to patients following an individual, picture, or object [45]. These results were confirmed by recent studies. VP was investigated in 51 MCS patients. Thirty-eight (75%) of them showed VP, and 11 (29%) only showed VP when a mirror was used [51]. Another study with 88 MCS patients investigated VP with different objects. VP was detected in 61/88 (69%) of patients, and in 16 (26%) of them VP was exclusively detected by a mirror [47].

VP was also studied in DOC patients quantitatively with an infrared eye tracker [48, 49]. Patients looked to either a moving red circle or a moving parrot, which were presented on a screen. VP was measured by electronically calculating the percentage of fixations on the target. MCS patients followed the target more frequent (32.9%) compared to UWS/VS patients (4.9%). In a second study from the same authors, a moving photo of a relative was added as an extra stimulus [49]. In MCS patients, a significant higher frequency of following the moving photo of a close relative was found (37.3%) compared to the images of the parrot (29.9%) and the circle (30.6%). In UWS/VS and healthy control subjects, no significant differences were seen between the applied stimuli [49].

### Assessment of visual fixation

Assessment of VF was discussed in five papers [50, 52–55] (Table 5). Different stimuli were discussed: objects like a mirror, a ball, a light [52], familiar photographs and a card [53–55]. In the WHIM, VF is mainly tested by looking at a person. In one reaction tested by the WHIM an object was used, but was not further specified. Two studies tested VF in combination with the techniques Brain Computer Interface (BCI) and functional Magnetic Resonance Imaging (fMRI), respectively [53, 55].

**Table 5:** Assessment of visual fixation

Author, year [references]	Assessment item	Population	Assessment method	Results	Conclusion of authors
Di et al. 2014 [52]	Use of mirror and/or ball and/or light	UWS/VS (*n* = 43) MCS (*n* = 38)	CRS-R	49% of total population showed VF (all MCS); 48% showed VF in response to mirror, 28% to ball, 25% to light	The frequency of VF in patients with DOC is related to the stimulus used. MCS patients tended to fixate significantly more on their own reflection compared to a brightly colored or illuminated object.
Pan et al. 2014 [53]	Subject’s own facial photo and an unfamiliar photo	Healthy Subjects (*n* = 4) UWS/VS (*n* = 4) MCS (*n* = 3) LIS (*n* = 1)	Visual hybrid brain computer interface CRS-R	Run 1: looking at own photo, accuracies in 5/8 patients (2 UWS/VS, 2 MCS, 1 LIS) Run 2: looking at unfamiliar photos, accuracies in 3/5 patients (1 UWS/VS, 1 MCS, 1 LIS) Run 3: looking at either own photo or unfamiliar photos, accuracies in 3/5 patients (1 UWS/VS, 1 MCS, 1 LIS), indicative of command following	Use of P300 and SSVEP BCI showed that VS, MCS and LIS patients looked accurately at either familiar or unfamiliar photos or to both photos.
Turner-Stokes et al. 2015 [50]	Fixation at individual or object	UWS/VS (*n* = 12) MCS- (*n* = 12) MCS + (*n* = 15) Emerged (*n* = 26)	WHIM item-by-item analysis	*Observed reaction* *State of consciousness* % Looks at person briefly UWS/VS UWS/VS: 14%; MCS-: 65%; MCS + 94% Makes eye contact (briefly) UWS/VS UWS/VS: 5%; MCS-: 35%; MCS + : 76% Looks at person giving attention UWS/VS UWS/VS 1%; MCS-: 36%; MCS + : 74% Looks at person talking to them (at least 3 s) UWS/VS UWS/VS: 1% MCS-: 32%; MCS + : 71% Maintains eye contact for 5 s and more: UWS/VS UWS/VS: 3%; MCS-: 28%; MCS + : 59% Looks at object when requested MCS minus UWS/VS: 0%; MCS-: 15%; MCS + 42% Seeks eye contact: MCS minus UWS/VS: 0%, MCS-: 6%; MCS + 37% Looks at and apparently explores pictures MCS minus UWS/VS: 0%; MCS-: 1%; MCS + : 27% Switches gaze spontaneously from one person to another MCS minus UWS/VS: 0%, MCS-: 1%; MCS + : 22%	No conclusion about VF^a^
Whyte and DiPasquale, 1995 [54]	Photos of patient’s family and plain white card	Minimally responsive patients (n = 6)	Photo and card presented in left/right visual field	Diagnosis on vision and visual attention Normal vision in both fields, monocular lesion, homonymous hemianopia left, homonymous hemianopia + possible impairment right eye, left sided extinction, right sided visual inattention	Single subject experimental protocols can be useful to assess vision and visual attention in minimally responsive patients since validated assessment methods are lacking
Zhu et al. 2009 [55]	Intimate family photos and pictures with emotional content from IAPS database	MCS (n = 9) Healthy controls (n = 10)	Family pictures, high- and medium stimulating pictures fMRI	Family pictures: 6/9 MCS patients show widespread activation in visual network, activation volume lower than in healthy subjects, but activation in network was similar High stimulating pictures: 2/9 MCS patients activation in visual network. Medium stimulating pictures 1/9 MCS patient activation in visual network	Pictures of family members with emotional valence, with which MCS patients were very familiar prior to their loss of consciousness, elicit greater activation of visual activity in the associated visual network

*BCI* brain computer interface, *IAPS* international affective picture system, *SSVEP* steady state evoked potential, *LIS* locked-in syndrome

^a^Data presented, no conclusion drawn about visual fixation reactions

Investigation of VF in MCS patients with different stimuli showed that VF was significantly more seen on the mirror (48%) compared to the ball (28%) and a light (25%) [52]. In an analysis of different items of the WHIM, maintaining gaze or gaze shifting reactions were more prevalent in MCS compared to UWS/VS patients [50].

Three studies discussed the use of visual stimuli with images of familiar persons. First, visual attention to a personal stimulus was compared to a neutral stimulus and patients oriented more frequent to the familiar image than to the neutral stimulus [54]. Second, in a BCI study, VF was investigated in patients with UWS/VS, MCS, locked-in syndrome and healthy controls. It was demonstrated that accuracies of attending to one’s own photo were higher than to unfamiliar photos. However, no differences between UWS/VS and MCS were found [53]. Third, an fMRI study investigated visual perception of different pictures in nine MCS patients and ten healthy controls [55]. In 6/9 MCS patients and all healthy controls looking at family pictures had higher activation in the visual networks compared to looking at other pictures.

### Influencing factors

Five influencing factors on visual responses were discussed in eight studies: within-day variability [56], inter-rater reliability (IRR) differences due to profession and/or experience [57, 58], presence of an informal caregiver [59], duration of assessment [60], and influences of visual/oculomotor impairments [6, 61, 62] (Table 6). Most of the results of these studies presented CRS-R visual subscale scores, which were not subdivided in VP and/or VF. First, visual subscale scores on the CRS-R were higher in the morning than in the afternoon, which could be explained by individual changes in visual functioning or by the presence of fragmentary cyclic processes [56]. Second, in two studies, IRR was investigated between different professionals and/or different levels of experience [57, 58]. The IRR on the visual subscale of the CRS-R was good (*k* = 0.73). The IRR of physicians was slightly higher (*k* = 0.81) compared to psychologists (*k* = 0.68) and a group of physiotherapists, speech therapists, and nurses (*k* = 0.73). Assessors who had > 24 months experience in assessing DOC patients showed a higher IRR (*k* = 0.81) than assessors with less experience (*k* = 0.62 for experience < 24 months and *k* = 0.68 for experience < 12 months) [57]. Another study showed a lower IRR for the visual subscale score of the CRS-R in experienced (*k* = 0.48) as well as in the less experienced assessors (*k* = 0.47) [58]. Third, the involvement of an informal caregiver in the assessment resulted in higher visual subscale scores on the CRS-R compared to assessment of a clinician alone [59]. Fourth, the duration of the assessment was investigated in 10 DOC patients. When two repeated assessment with the CRS-R (50–60 min) and 10 SMART assessment (600 min) were compared, this led to differences in the level of consciousness in 4/10 patients. In 2/4 patients, these differences were caused by detecting sustained VF with the SMART and not with the CRS-R [60]. Fifth, influences of visual impairments and/or oculomotor defects on assessment of the level of consciousness were found in 3 studies [6, 61, 62]. In misdiagnosed UWS/VS patients, 65% had visual impairments [6], and in MCS patients, 9/52 (17%) scored no visual responses on the CRS-R [62] and analysis of CRS-R subscale scores showed that visual problems such as optical nerve damage, ptosis, ocular apraxia and visual agnosia could cause improbable CRS-R scores [61].

**Table 6:** Influencing factors on visual responses

Author, year [References]	Factors	Population	Assessment method	Results of authors	Conclusion of authors
Andrews et al. 1996 [6]	Visual impairment/blindness	UWS/VS (*n* = 40) admitted 1992–1995	Diagnosis derived from medical records	17/40 (43%) were misdiagnosed, 11/17 (65%) were blind or severely visually impaired	The very high prevalence of visual impairment is a complicating factor since physicians making a diagnosis of the vegetative state place great emphasis on the inability to visually track or blink to threat
Chatelle et al. 2016 [61]	Oculomotor defects	DOC (*n* = 1190); UWS/VS (*n* = 464) MCS (n = 586)	CRS-R	*Oculomotor factors and improbable CRS*-*R scores*^a^ Ptosis or eye lid apraxia: VF/unarousable Bilateral optic nerve damage, Terson syndrome, cortical blindness: No visual response/consistent command following No visual response/functional communication Third and fourth cranial nerve palsy, ocular apraxia, visual agnosia: VF/functional communication VP/functional communication	CRS-R scores are subject to attributable inaccuracy of examiner error and other confounding that can lead to misinterpretation of results
Cortese et al. 2015 [56]	Variation during the day	UWS/VS (*n* = 7) MCS (*n* = 12)	CRS-R	CRS-R visual subscale higher in the morning than in the afternoon	CRS-R differences between morning and afternoon are likely to reflect individual changes in patient’s visual, auditory and motor conceivably due to changes in neuronal/non neuronal factors that modulate the brain state
Estraneo et al. 2015 [57]	Profession and experience of assessors	DOC (*n* = 27)	CRS-R	IRR CRS-R visual subscale All raters *k* = 0.73 Physicians *k* = 0.81 Psychologists *k* = 0.68 Nurse/physiotherapist/speech therapist *k* = 0.73 Expertise high (> 24 months) *k* = 0.81 medium (12-24 months) *k* = 0.62 low (< 12 months) *k* = 0.68	Results did not change as a function of professional specialities or experience Good IRR was found for all subscales, especially for the visual subscale
Estraneo et al. 2015 [62]	Oculomotor defects	MCS (*n* = 52)	CRS-R	9/52 MCS patients could not produce non-reflexive movements in the visual subscale	The visual subscale of he CRS-R could misdiagnose as UWS/VS as MCS patients with oculomotor defects could not produce non-reflexive responses on the visual subscale
Godbolt et al., 2012 [6]	Duration of assessment	DOC (n = 10)	CRS-R SMART	In 4/10 differences in diagnosis between CRS-R (2 assessments of 50 min) and SMART (10 assessments of 60 min). 2 additional MCS diagnosis based on visual fixation and visual tracking	Brief behavioural assessment is not as effective as extended assessment in detecting non-vegetative behaviours. Total time spent in behavioural assessment is likely important
Lovstad et al. 2010 [58]	Experience of assessors	DOC (*n* = 31)	CRS-R	IRR experienced raters *k* = 0.46 IRR l less experienced raters *k* = 0.47 TRR experienced raters *k* = 0.86 TRR less experienced raters *k* = 0.57	The auditory and visual subscales might be most susceptible to interrater disagreement for less experienced raters.
Sattin et al. 2014 [59]	Presence of informal caregiver	DOC (*n* = 153) UWS/VS 53% MCS 40%	CRS-R	Significant difference in visual subscale between CRS-R done by rater alone and CRS-R done by rater + informal caregiver (effect size Cohen’s D 0.33). Visual subscale scores were higher in assessments of rater + informal caregiver in MCS and severe disability compared to UWS/VS	Informal caregivers can increase capacity of raters to detect visual responses Visual stimuli furnished by familiar persons could be more attractive

Visual responses not further specified

^a^Only improbable combinations displayed in which VP or VF or absence of visual responses was involved

## Discussion

To our knowledge, this is the first review that addresses the question whether or not VP and VF are related to consciousness. We found that literature about the importance of these responses in relation with consciousness still is controversial. No agreed-upon definition of VP and VF was found and the assessment methods vary widely regarding procedures and operational criteria. However, the studies generally agreed that the use of a mirror is the most sensitive method to detect VP and VF.

The lack of an agreed-upon definition has led to international differences in interpretations. In the United States, VP and VF are considered signs of MCS, whereas in the United Kingdom (UK) these signs are atypical but viewed as signs of UWS/VS [63–65]. In addition, not operationally defined terms like ‘brief’ and ‘sustained’ VP and/or VF, can cause differences in interpretation with a risk of diagnostic inaccuracy. Furthermore, a recent expert opinion stated that there is no rationale why a brief visual response does not require consciousness and a sustained response does [66]. To conclude, evidence for the use of ‘brief’ and ‘sustained’ VP and VF for distinguishing UWS/VS from MCS is lacking.

A wide variety of assessment methods with variable operational criteria of VP and VF were found. Only the CRS-R and the STAR were developed with a diagnostic purpose. The other scales were mainly developed to monitor neurobehavioral functions. Judging the validity of the different scales is difficult because a golden standard is lacking for diagnosis of the level of consciousness. In 2010, 13 DOC assessment scales were reviewed. The CRS-R is the only scale recommended with ‘minor reservations’ because it has acceptable administration and scoring guidelines and good content validity. Despite the recommendations for clinical use, the authors of this review stated that evaluation of diagnostic validity remains problematic. Diagnostic validity was unproven for all assessment scales and interpretation is difficult because of the lack of a standard criterion measure for the assessment of the level of consciousness [67].

The use of a mirror appeared to be the most sensitive method to detect VP and VF [47, 51, 52]. It has been suggested that the use of patient’s own face can be useful to detect residual self-awareness [68] and that personally relevant stimuli increase the probability of detecting a conscious response in DOC patients [69]. However, recent studies published after our search period indicate that the sensitivity of the mirror cannot be explained by a lower cognitive demand [70], neither the self-referential aspect of the mirror is viewed as a complete explanation [71]. Therefore, the rationale for the sensitivity of the mirror has not been clarified yet.

The absence of visual responses in a considerable part of the DOC patients calls for a nuance to the view that VP and VF are important signs for detecting consciousness. Although it was demonstrated that visual responses were the signs most frequently detected in MCS patients, the absence in about 20% of the MCS population cannot be ignored [62]. Examination of the integrity of the visual tract with techniques like visual evoked potentials and imaging is advisable in patients with DOC who do not show visual responses. A closer look into the neurobiology of VP and VF shows that VP is considered to be under volitional control [72]. For VF, however, it remains questionable if this sign is a conscious response because saccadic eye movements are necessary to shift gaze from one position to another. Saccades can be either voluntary or reflexive [73, 74]. The existence of accurate localization in the visual field without consciously processing visual stimuli, which can be present in patients with blindsight and visual form agnosia, further complicates the understanding of the association of VP and VF with consciousness. Since the association of VP and VF with consciousness remains questionable, further research is needed. Longitudinal studies which follow VP and VF during the recovery phase may give insight into the question if and/or how VP and VF are associated with consciousness.

There are some limitations regarding the literature search and the interpretation. First, the methodological quality of the included papers was not systematically assessed. Because we included theoretical, empirical and expert-opinion papers, a uniform quality assessment was not possible. Second, different descriptions that existed for VF such as ‘focusing on the examiner’ and ‘active looking for objects’, might have led to possible misinterpretation of these reactions as VF. Third, the SMART might be a proper scale for assessment; however, we could not evaluate the properties of this scale, since it requires formal training and it must be purchased. Previously, it has also been reported that the SMART may not be accessible for users outside the UK [67].

In conclusion, the question whether or not VP and VF are signs of MCS cannot be answered uniformly yet. This review demonstrates a lack of consensus regarding definition, operationalization and assessment methods. Although VP and VF are widely recognized as signs of emerging consciousness, the supporting evidence is scarce. Moreover, since VP and VF are included into the diagnostic criteria of MCS, it is not surprising that these signs are more prevalent in MCS patients than in UWS/VS patients. One can speak of a circular argument if based on such a prevalence difference, authors conclude that VP and VF are indicative of consciousness. More research is needed to investigate the validity of these signs to measure the level of consciousness before adopting them as important diagnostic signs of MCS. Therefore, we recommend international consensus development about definitions, operational criteria and assessment procedures of VP and VF. Reaching consensus about these first signs of consciousness is highly important for a proper diagnosis and consequently increases the chance for providing rehabilitation to this population. As recently stated by Fins [75], misdiagnosis of MCS patients as UWS/VS, can deny them access to rehabilitation and thereby marginalizes these patients. Proper identification of MCS can pave the way for rehabilitation and thereby breaching the marginalization of these vulnerable patients.

## Electronic supplementary material

Below is the link to the electronic supplementary material.

Supplementary material 1 (DOCX 1740 kb)

## Footnotes

1 We will use the term visual pursuit. When we refer to a specific paper, we will use the terminology mentioned in that paper.
