# Supplementary material for: Are visual functions diagnostic signs of the minimally conscious state? an integrative review
Source: J Neurol. 2018 Feb 28;265(9):1957–75. doi: 10.1007/s00415-018-8788-9 (PMC6132665; doi:10.1007/s00415-018-8788-9)
Supplement: Supplementary file 1 — Supplementary material 1 (DOCX 1740 kb) [file 415_2018_8788_MOESM1_ESM.docx]

**Supplement 1** Complete Query

((("minimally conscious" OR "Persistent Vegetative State"[Mesh]) AND (Diagnosis/Broad[filter] OR prognosis OR assess OR assessment OR misdiagnos*))) OR (((((("Persistent Vegetative State"[Mesh]) OR (((("Consciousness Disorders"[Mesh:noexp]) OR "Unconsciousness"[Mesh:noexp]) OR "Coma"[Mesh:noexp]) OR "Coma, Post-Head Injury"[Mesh]))) OR minimally conscious*) OR ((minimally responsive state*) OR (minimally responsive AND patient* OR low functioning head injured OR (coma AND recovery)))) AND (((((("Vision Disorders"[Mesh:noexp]) OR "Eye Movements"[Mesh]) OR "Visual Perception"[Mesh:noexp]) OR "Pursuit, Smooth"[Mesh]) OR "Fixation, Ocular"[Mesh]) OR (eye tracking OR visual tracking OR pursuit OR fixation) OR visual behavioral responses))
